# An uncommon variant of Alzheimer’s disease: Posterior cortical atrophy

**DOI:** 10.4102/sajpsychiatry.v31i0.2420

**Published:** 2025-05-23

**Authors:** Tasneem Bux, Kyle Pillay, Reyna Daya, Zaheer Bayat, Lara S. Greenstein

**Affiliations:** 1 Department of Internal Medicine, Faculty of Health Sciences, University of the Witwatersrand, Johannesburg, South Africa; 2 Division of Geriatric Medicine, Faculty of Health Sciences, Wits Donald Gordon Medical Centre, Johannesburg, South Africa; 3 Division of Diabetes, Endocrinology and Metabolism, Faculty of Health Sciences, Helen Joseph Hospital, Johannesburg, South Africa; 4 Division of Geriatric Medicine, Faculty of Health Sciences, Helen Joseph Hospital, Johannesburg, South Africa

**Keywords:** posterior cortical atrophy, Alzheimer’s dementia, visuospatial apraxia, neurodegeneration, atypical Alzheimer’s

## Abstract

**Introduction:**

This case report highlights one of the less common variants of major neurocognitive disorder because of Alzheimer’s disease, posterior cortical atrophy. There is a paucity of data about this condition in sub-Saharan Africa.

**Patient presentation:**

A 54-year-old female presented to the geriatric clinic following a 2-year history of poor memory and inability to fulfil her work obligations. The most prominent symptom was visual disturbance, with a normal ophthalmic examination.

**Management and outcome:**

Workup done to reveal reversible causes of dementia did not yield any positive results. After a full history, physical and cognitive examination and radiological investigations, a diagnosis of posterior cortical atrophy variant of major neurocognitive disorder because of Alzheimer’s disease as the most likely aetiology was established.

**Conclusion:**

Posterior cortical atrophy is a rare variant of Alzheimer’s disease, and no case reports from South Africa are available in the literature.

**Contribution:**

This case reminds us that unusual presentations of cognitive impairment require a broad differential diagnosis.

## Introduction

Posterior cortical atrophy (PCA) is an uncommon progressive neurodegenerative syndrome characterised by atrophy of the occipitoparietal cortices, leading to visuospatial impairment, visual processing abnormalities and apraxia.^1^ Neurodegeneration is usually because of underlying Alzheimer’s disease (AD), but has been associated with dementia with Lewy bodies (DLBs), corticobasal degeneration (CBD) and prion disease.^2,3^ Posterior cortical atrophy shares features of both Gerstmann and Balint syndrome, which are other less common neurocognitive disorders.^4,5,6^

Differences between PCA and typical AD include a younger age of onset, visual symptoms, marked visuo-spatial deficits and later involvement of memory and language problems, while similarities include the insidious onset and gradual progression. The APOE e4 allele may be present, although less commonly than in typical amnestic AD. Patients with PCA are often misdiagnosed with anxiety or depression early in the course of the disease.^5,6^ Because of the atypical presentation, diagnosis is often delayed, with patients initially seeing ophthalmologists years prior to consulting neurologists, psychiatrists or geriatricians.^2,3,5^ The diagnosis is made clinically with supportive radiological findings.^4^

The prevalence of PCA is unknown, but it is estimated that 5% of those diagnosed with AD may have PCA and may be as high as 13% in those with early-onset AD.^7,8,9^ In 2017, new three-level consensus criteria were published for the diagnosis and classification of PCA.^10^ The need for the sparing of anterograde memory function, executive function as well as speech and non-visual language function means that patients are often not considered to fulfil the Diagnostic and Statistical Manual for Mental Disorders (DSM) V criteria for major neurocognitive disorder (MNCD) because of AD until later in the disease process. To differentiate between the potential underlying aetiologies, the level 2 classification of PCA pure versus PCA plus must first be determined; PCA plus is more likely to be because of underlying aetiology other than AD. There should be no biomarker evidence of AD for PCA-Lewy body disease (LBD) or PCA-prion. In a fully resourced setting, the diagnosis of PCA-AD requires the fulfilment of PCA syndrome plus in vivo evidence of AD pathology: decreased amyloid beta 1-42 or increased total Tau in cerebrospinal fluid (CSF), increased tracer retention on amyloid positron emission tomography (PET) and the presence of autosomal dominant mutation for AD.^6,7,9^ There is no data on early-onset AD or PCA, nor any case reports from South Africa.

### Ethical considerations

Ethical clearance to conduct this study was obtained from the University of the Witwatersrand Human Research Ethics Committee (Medical) (No. M220791). The patient gave written consent.

## Patient presentation

### Case

A 54-year-old African female domestic worker attended the geriatrics outpatient clinic, with a 2-year history of poor memory and an inability to complete her everyday tasks. Her symptoms began with slowed work performance, eventually progressing to difficulties using household appliances and frequently dropping items. She experienced ‘tunnel vision’. She previously consulted a general practitioner, optometrist and an ophthalmologist. Depressive symptoms were absent, and the neuropsychiatric inventory did not reveal any delusions, hallucinations, agitation or disinhibition. She did display features of irritability and apathy, but this was neither severe nor disruptive. She had no background medical problems or family history of cognitive or neurological disorders.

Physical examination revealed a well-looking female. A previous optometry evaluation revealed dry eyes, good distance vision and presbyopia. She had difficulty focussing on a static target and had delayed and erratic tracking. She had no tremor, asymmetrical rigidity or bradykinesia. She did not show focal neurological signs and did not have upgoing plantar reflexes. She was unable to complete tasks she had previously done in her employment, such as packing the dishwasher and folding clothes. She could no longer use the washing machine or fasten her seatbelt. This was in keeping with a motor apraxia.

**FIGURE 1:**
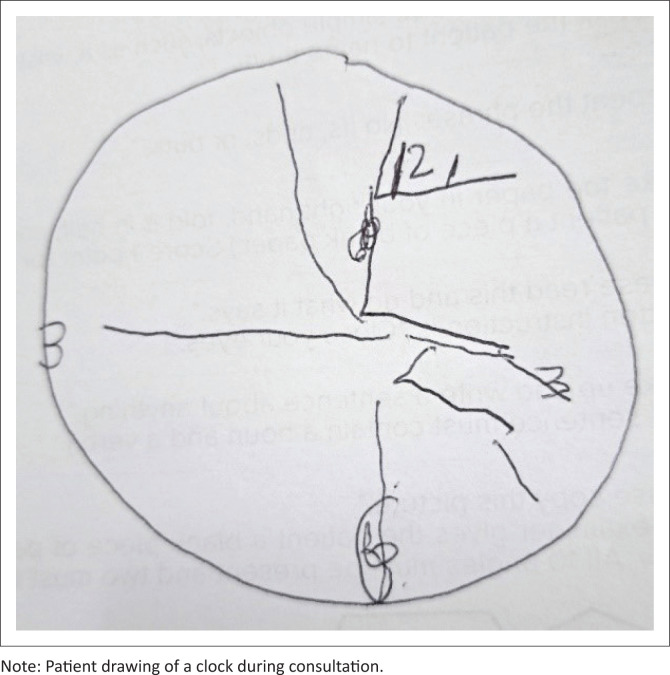
Clock-drawing test.

The MMSE (mini-mental state examination) score was 21 out of 30 with points lost on orientation, serial 7’s, delayed recall and language and visuo-constructive parameters. Her son had to take over instrumental activities of daily living for her, such as cooking, managing her finances and helping her with shopping. The clock-drawing test showed grossly abnormal visuospatial deficits (see Figure 1). Blood tests and CSF evaluation to rule out common reversible causes were normal. In our setting, we were unable to test for blood or CSF biomarkers of amyloid pathology.

**FIGURE 2:**
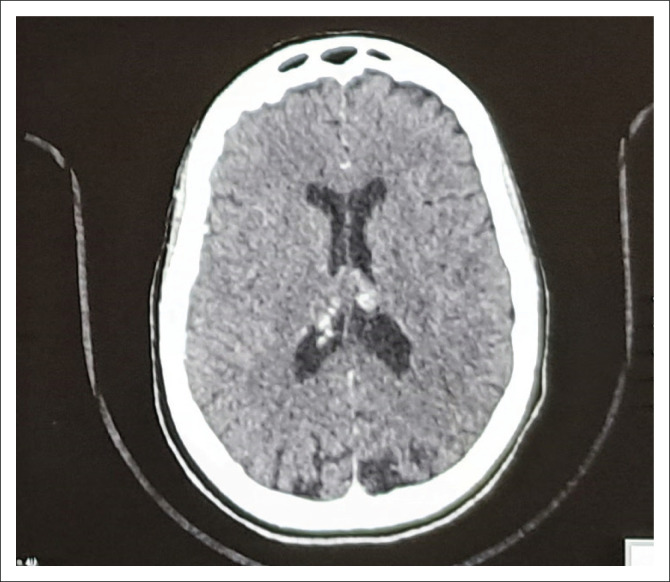
A post-contrast computed tomography scan of the brain showing brain atrophy most prominent posteriorly, in keeping with this diagnosis.

A provisional diagnosis of PCA variant of Alzheimer’s dementia was made with computed tomography (CT) scan imaging showing brain atrophy most prominent posteriorly, in keeping with this diagnosis (see Figure 2). Magnetic resonance imaging (MRI) would have been the preferred imaging modality but was temporarily unavailable at the time of presentation. Nuclear medicine studies, such as an amyloid PET, which may show deposition of amyloid within the occipital cortices, are also helpful, but this level of imaging was not available in our setting.

As this condition is incurable and progressive, the patient received counselling. She received a disability grant application form, and she chose to relocate to the Eastern Cape for family support. Because of the rural location of her family home, she declined further referral to support services. Helpful further services would include occupational therapy, environmental adaptation and counselling services for herself and her family.

## Discussion

Crutch et al. published revised consensus criteria for the diagnosis of PCA in 2017, and our patient fulfils these criteria in the following aspects:^4,10^

- Her condition was of an insidious onset with gradual progression over 2 years.
- She demonstrated space perception and object deficit, and constructional apraxia from the onset of her symptoms.
- Her anterograde memory, speech and language, executive function and behaviour were relatively spared.
- There was no evidence of a brain tumour, significant vascular disease, evidence of afferent visual cause or other identifiable causes of cognitive impairment.

Neuroimaging shows predominant atrophy in the occipitoparietal or occipitotemporal regions, often seen as areas of hypometabolism or hypoperfusion on nuclear imaging. Amyloid positron emission tomography (PET) may identify underlying AD pathology, 18 fluorodeoxyglucose positron emission tomography (FDG PET) may support the diagnosis by demonstrating the characteristic pattern of hypometabolism, while single-photon emission computerised tomography (SPECT) may reveal abnormal dopamine transport. We were unable to access these imaging modalities in our setting, nor were we able to assess CSF or blood for biomarkers, such as the presence of Tau protein in the CSF or positivity for APOE allele.^9,11^ Cerebrospinal fluid examination may be positive for biomarkers of AD or prion disease, and amyloid biomarkers may be present; however, no biomarkers are specific to the clinical syndrome of PCA.^3,5,11,12^

There is no cure for PCA. Disease-modifying therapies are not available; cholinesterase inhibitors have been used in the management of PCA. Selective serotonin reuptake inhibitors have been used in the management of associated depression and anxiety, melatonin may be considered for sleep disturbances and low dose atypical antipsychotic agents may be used with caution in those who develop behavioural or psychiatric symptoms. The mainstay of treatment remains non-pharmacological, with options including occupational therapy with a focus on interventions that compensate for visual limitations, environmental modifications and the provision of education and psychosocial support to patients and their caregivers.^4,12,13^

In their retrospective study, Sanli et al. found depression in 40% of patients.^14^ Depression in those with PCA, and indeed other MNCDs, presents a diagnostic dilemma as it can masquerade as a pseudo-dementia.^15^ The depression may result from preservation of insight early in the disease. Hotz et al. described the case of a 65-year-old male with PCA who had been misdiagnosed with major depressive and generalised anxiety disorder.^15^ This patient had mood symptoms with episodes of suicidal ideation.

Genetic risks from a genome-wide association study (GWAS) of AD and DLB identified PCA risk association at known AD risk loci, including APOE, but not at the two DLB loci tested. Picillo et al. also identified a variant of glucocerebrosidase (GBA), a genetic risk factor for Parkinson’s disease and DLB, in a patient in which neuroimaging was suggestive of PCA.^10,16^

## Conclusion

Posterior cortical atrophy is a rare and often misdiagnosed neurodegenerative syndrome. Younger age of onset and selective visual processing decline, attributable to posterior cortical dysfunction, should prompt consideration of PCA. Non-pharmacological management remains the mainstay of treatment as disease-modifying therapies are unavailable. Data on early-onset AD and PCA in South Africa are scarce, highlighting critical research needs.
